# Saliva-Based ELISAs for Effective SARS-CoV-2 Antibody Monitoring in Vaccinated Individuals

**DOI:** 10.3389/fimmu.2021.701411

**Published:** 2021-09-03

**Authors:** Joseph G. Casian, Aaron N. Angel, Ronell Lopez, Cedie Bagos, Melanie A. MacMullan, Mindy L. Bui, Prithivi Chellamathu, Sudipta Das, Fred Turner, Vladimir Slepnev, Albina Ibrayeva

**Affiliations:** ^1^Department of Serology Research and Development, Curative, Monrovia, CA, United States; ^2^Mork Family Department of Chemical Engineering and Materials Science, Viterbi School of Engineering, University of Southern California, Los Angeles, CA, United States; ^3^Eli and Edith Broad Center for Regenerative Medicine & Stem Cell Research at the University of Southern California, Department of Stem Cell Biology and Regenerative Medicine, W.M. Keck School of Medicine, Los Angeles, CA, United States; ^4^Davis School of Gerontology, University of Southern California, Los Angeles, CA, United States

**Keywords:** SARS-CoV-2, immunology, saliva-based antibody detection, ELISAs, vaccines, diagnostic

## Abstract

In March 2020, the World Health Organization (WHO) declared a global health emergency—the coronavirus disease 2019 (COVID-19) pandemic. Since then, the development and implementation of vaccines against the virus amidst emerging cases of re-infection has prompted researchers to work towards understanding how immunity develops and is sustained. Serological testing has been instrumental in monitoring the development and persistence of antibodies against SARS-CoV-2 infection, however inconsistencies in detection have been reported by different methods. As serological testing becomes more commonplace, it is important to establish widespread and repeatable processes for monitoring vaccine efficacy. Therefore, we present enzyme linked immunosorbent assays (ELISAs) compatible for antibody detection in saliva as highly accurate, efficacious, and scalable tools for studying the immune response in individuals vaccinated against SARS-CoV-2.

## Introduction

In December 2019, a novel coronavirus emerged, causing widespread respiratory illness and earning the name Coronavirus Disease 2019 (COVID-19) (1). In the months that followed, the SARS-CoV-2 virus rapidly spread from a series of cases in the Wuhan province of China resulting in a global pandemic, infecting millions worldwide. Many infected individuals have had minor or no symptoms, which contributed to high transmissibility by allowing the virus to spread undetected from low-symptomatic and asymptomatic individuals to others. 

**Figure 1:**
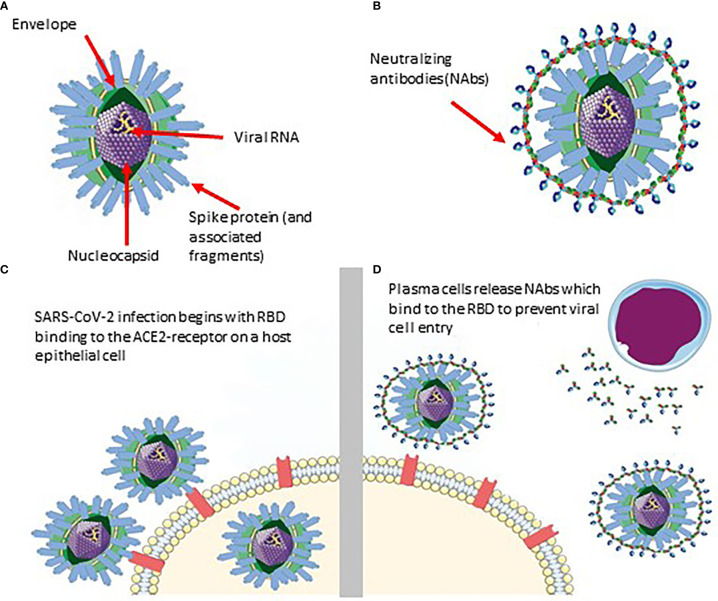
Schematic of SARS-CoV-2 infiltration and neutralization. **(A)** SARS-CoV-2 viral structure. **(B)** Neutralizing antibodies developed against SARS-CoV-2. **(C)** Infiltration of SARS-CoV-2 into the host cell via RBD binding. **(D)** Neutralizing antibodies bind specifically to a SARS-CoV-2 epitope (RBD in this case) to prevent it from entering the epithelial cell.

Researchers have shown that previously infected individuals develop SARS-CoV-2 specific antibodies which persist for at least 8 months post-infection (**Figure 1**) (2). Immunoglobulin G (IgG), with respect to two other antibodies present (IgM and IgA), is known to maintain stability and neutralizing activity in serum for several months following symptom onset, providing a proxy for monitoring long-term immune response (3–5). Therefore, antibody testing can permit the tracing of viral spread post-infection as long as antibodies persist in previously infected individuals. 

Furthermore, reliable antibody testing will become increasingly useful for tracking vaccine efficacy and the development of herd immunity in our population. With the current vaccination rollout, an efficient, effective, and easily implemented serological assay will be essential for ensuring a safe return to pre-pandemic normalcy. Here, we describe ELISAs that have been studied for the detection of antibodies against SARS-CoV-2 and discuss their potential as optimal tools for monitoring the development of herd immunity within the population.

## Vaccines and Vaccine Development

As immunizations are beginning to become widely administered and available, it is important to implement a universal test that will allow us to monitor and confirm the development of an immune response against SARS-CoV-2. The main objective for all the major vaccines against SARS-CoV-2 is to elicit an immune response which can in turn protect people from severe disease and mortality. Secondary to that goal is to reduce the transmissibility of SARS-CoV-2 in the population, thereby reducing the number of new variants. Long-term SARS-CoV-2 antibody detection is thus crucial to determining the durability of the humoral response following vaccination.

Initial clinical studies from the Kaiser Permanente Washington Health Research Institute on 45 individuals receiving the Moderna vaccine have indicated that the vaccine elicits both binding and neutralizing antibody responses that develop approximately two weeks after vaccination (6). Studies on mRNA vaccines developed from both Moderna and Pfizer have found that the S-protein binding IgG concentrations were higher than those from convalescent plasma donors who acquired endogenous SARS-CoV-2 antibodies (7). Short-term interim results from clinical studies at the National Institute for Allergies and Infectious Diseases on 34 patients acquiring antibodies after receiving the Moderna vaccine show that the humoral response remained robust 119 days after receiving a complete dose of the mRNA-1273 vaccine (8). Additionally, Sadoff et al. showed that antibody presence and neutralizing capability following the Moderna and Johnson and Johnson vaccines were strongly correlated (9). Given the expected need to test mass populations rapidly, accurately, and safely on a long-term monthly basis to evaluate the presence and persistence of SARS-CoV-2 antibodies, an assay which couples rapid specimen collection with high-throughput processing and analysis would be optimal for universal monitoring.

## Immunoassays

ELISAs are often implemented in a well-plate format, making them easy to automate and scale for high-throughput screening of antibody response developed against SARS-CoV-2 infection. ELISAs for detection of SARS-CoV-2 antibodies incorporate peptide fragments of the virus itself, including the nucleocapsid (N) protein, the spike (S) protein, and the receptor binding domain (RBD). Antibodies from biofluids, such as blood, serum, or saliva, that bind to the antigens are detected through a second incubation step. If bound, an enzyme-labeled anti-human antibody reacts with a substrate to produce a color indicating the presence of antibodies (**Supplementary Figure 1**). We review here some commercially developed ELISA kits which have received Emergency Use Authorization (EUA) by the FDA as well as several kits developed in-house by a number of research groups (**Table 1**) (10). Kits developed in-house have the advantage of being less expensive and more accessible to researchers, thus studies involving in-house ELISAs typically have larger study cohorts.

**Table 1:** Summary and statistics of reviewed EUA-approved, non-EUA approved, and in-house ELISA kits.

Test Kit	Sensitivity (%)	Specificity (%)	Target	N
Anti-SARS-CoV-2 IgG ELISA (Euroimmun)	90.0	100	Spike (S) protein	30
Anti-SARS-CoV-2 IgA ELISA (Euroimmun)*	86	92	S Protein	57
OraSureTechnologies Oral Fluid Specimen ELISA*	100	100	S1 fragment of S Protein	147
Beijing Wantai Biological Pharmacy Enterprise WANTAI SARS-CoV-2 Ab ELISA	96.7	97.5	S Protein	30
NovaLisa Anti-SARS-CoV-2 ELISA*	35.5 for IgG 19.4 for IgM 45.2 for IgG + IgM		Nucleocapsid (N) Protein	40
Gold Standard Diagnostics Anti-SARS-CoV-2 ELISA*	69 for IgG 15 for IgA	100	N Protein	123
Mass General Hospital and Harvard Medical School Anti-RBD antibody in house ELISA*	95 for IgG 90 for IgA 81 for IgM	100	Receptor Binding Domain (RBD) of S Protein	343
Mount Sinai IgG Anti-SARS-CoV-2 in house ELISA*	92.5	100	S Protein	120
University of Toronto Anti-SARS-CoV-2 in house ELISA*	95.5 for S Protein 91.3 for RBD		S Protein, RBD	439

*These ELISA kits have not received an EUA approval

### Serum-Based ELISAs

Researchers from the Odense University Hospital in Denmark evaluated the performance of 6 commercially available SARS-CoV-2 antibody detection assays, two of which utilized ELISA (11). Both EUROIMMUN IgG and Wantai IgM kits were evaluated using 57 previously SARS-CoV-2 positive individuals and 200 pre-COVID blood donation specimens. Both the EUROIMMUN SARS-CoV-2 antibody detection kits and the Wantai IgM ELISA kit incorporate the RBD from the SARS-CoV-2 S protein as the assay antigen. The Wantai IgM ELISA detected antibodies in 79% of participants, while the EUROIMMUN ELISA had 96.2% sensitivity for detecting IgG antibodies.

A study from the University of Aix-Marseille in France also evaluated the efficacy of the EUROIMMUN IgG kit as well as the commercially available NovaLisa IgG and IgM kits using serum contributed by 40 individuals previously infected by SARS-CoV-2 and 10 individuals who had not been exposed to the virus (12). The NovaLisa ELISA employs an antigen from the N protein of SARS-CoV-2. This group found the performance of the EUROIMMUN IgG kit to be weaker than was previously suggested, with a sensitivity of 61.3%. They also found the sensitivity of the NovaLisa IgG kit to be less than 50% and estimated the sensitivity of the NovaLisa IgM kit to be between 19.4% and 35.5%.

In a comparative study, earlier work from our group evaluated the performance of IgG and IgA kits developed both by EUROIMMUN and Gold Standard Diagnostics (GSD) on serum collected both from 123 symptomatic and previously SARS-CoV-2 PCR-positive individuals and 83 PCR-negative individuals (13). The GSD ELISA is an adaptation from the NovaLisa ELISA which also uses an antigen from the N protein of SARS-CoV-2. We found IgG and IgA kits from GSD to have 100% specificity as well as 69% and 15% sensitivity, respectively. Additionally, we found the EUROIMMUN IgG and IgA kits to have 100% and 92% specificity and 90% and 86% sensitivity, respectively. We believe the discrepancy in performance between our study and the study from the University of Aix-Marseille is attributable to the difference in sample size illustrating limitations of both specimen collection for ELISA processing and reliance on a manufacturer to provide commercially developed plates. These discrepancies illustrate a need for ELISAs that can be developed in-house with reliable quality control, as we describe below.

After developing their own ELISA using an antigen from the RBD of the S protein, Iyer et al. from Mass General Hospital and Harvard Medical School evaluated the development and persistence of IgG, IgA and IgM antibodies in 343 participants previously infected by SARS-CoV-2 for up to 122 days post symptom onset. These researchers found their assay to have 100% specificity and sensitivities of 95% for IgG, 90% for IgA, and 81% for IgM antibodies (3). When compared to serum collected pre-pandemic from 1548 individuals, this group found that serum-based antibody concentrations dropped below their established positive threshold for IgM around 30 days and around 70 days for IgA. In addition, IgG levels persisted above the pre-pandemic controls throughout their study.

Another research group from the Icahn School of Medicine at Mount Sinai monitored the development and persistence of neutralizing antibodies in more than 30,000 previously infected individuals. These individuals confirmed the persistence of neutralizing antibodies through evaluating the correlation between their neutralizing assay and the “Mount Sinai ELISA”, developed in-house (4). The Mount Sinai ELISA was specific to IgG antibodies and was found to have 92.5% sensitivity and 100% specificity. This assay was used to perform antibody quantification by establishing a baseline of 120 serum samples with known ELISA titers. Over a period of 5 months, Wajnberg et al. found that antibodies against SARS-CoV-2 were detectable at relatively stable titers both by their in-house ELISA and neutralizing antibody assay.

These studies have used serum to evaluate antibody prevalence post infection by SARS-CoV-2 and to test the performance of various ELISA kits. Accordingly, ELISAs developed in-house have the potential to meet or exceed the performance of commercially available assays. Furthermore, studies which use assays developed in-house are more affordable and readily available, enabling them to be used to screen more individuals. However, despite the success of many of these studies in performing serum-based ELISAs for SARS-CoV-2 antibody detection, there are a number of drawbacks which result in a lack of accessibility and affordability.

Blood serum is derived from whole blood, meaning that a trained phlebotomist must perform the blood collection and serum isolation from a participant. This process is time-consuming, costly, and puts the phlebotomist in direct contact with the patient, presenting a health risk. While fully automated ELISAs can be compatible with the need for high-throughput screening, the inability to collect samples in a rapid, high-throughput way results in a bottleneck for specimen analysis. Another biofluid that could be collected without the requirement of a trained personnel is saliva, or oral fluid. It has been demonstrated, as we will describe in more detail, that saliva-based assays for antibody detection, and particularly for SARS-CoV-2 antibody detection, perform similarly to serum-based assays while eliminating the challenging sample collection barrier compared to the use of serum.

### Saliva-Based ELISAs

There are two major antibodies that can be detected in oral fluids: secretory IgA (SIgA) and IgG. While SIgA is produced locally in the salivary glands, most of the IgG in saliva comes from antibodies that are produced in the serum and cross into saliva through gingival crevices in the gums. Recently published longitudinal data tested paired saliva and serum samples in 402 convalescent patients confirmed to have COVID-19 through rt-PCR and 339 pre-COVID samples. The results showed that the detection of antibodies in the saliva, primarily IgG, correlated to levels of antibodies in the serum using an in-house developed saliva-based ELISA. More interestingly, IgG was detectable in saliva up to 105 days in both serum and saliva. The levels of IgA and IgM, which typically are the first two major antibodies to decline in serum post infection, also decayed in saliva. The ELISA that was developed for measuring IgG had a sensitivity of 95.6% using spike protein and 93.8% using RBD (14). This sensitivity is comparable to EUA approved serum-based ELISA kits for detecting antibodies against SARS-CoV-2 and this study demonstrates the importance and adequacy of saliva-based specimens for the detection of antibodies against SARS-CoV-2.

Although blood serum has been traditionally used in ELISAs, there has been a noted interest prior to the onset of the COVID-19 pandemic in developing an ELISA that can use a saliva sample. In an effort to improve the quality of specimens and testing procedures compared to rapid diagnostic tests, Beelaert et al. sought to validate and assess the usefulness of two oral fluid ELISAs for the detection of HIV antibodies (15). Using 140 oral fluid specimens (Intercept Oral Specimen Collection Device, OraSure Technologies, Bethlehem, USA) from HIV positive patients, the researchers found the Genscreen™ HIV-1/2 (Bio-Rad, Marnes-La-Coquette, France) and adapted Vironostika HIV Ag/Ab (Biomérieux, Marcy L'Etoile, France) ELISAs to have sensitivities of 100% and 95.7% as well as specificities of 97.3% and 100%, respectively. A saliva-based ELISA has also been developed and validated to measure IgG antibodies in response to human T-lymphotrophic viruses type 1 and 2 (HTLV-1/2). Woo et al. tested paired plasma and oral fluid (Oracol device, Malvern Medical Developments, Worcester, UK) of HTLV-1/2-seropositive patients with an in-house ELISA (n = 131) and a randomly selected subset of patients (n = 36) with the commercially available Murex HTLV I+II EIA (DiaSorin, Dartford, UK) (16). They found their in-house ELISA to be 100% sensitive and specific for both specimen types. The 36 oral fluid samples run with Murex HTLV I+II EIA for comparison yielded a lower sensitivity (86%) and 100% specificity. However, the 5 of 36 nonreactive samples also displayed low reactivities in the in-house ELISA and this commercial assay is configured solely for the analysis of serum or plasma. In addition, they found a strong correlation between the paired oral fluid and plasma signal/cutoff values from their in-house ELISA, further demonstrating oral fluid as an alternative to venous blood for serosurveillance of infectious diseases.

Oral fluid antibody prevalence has also been shown to be a valuable tool for evaluating vaccine campaigns. Nigatu et al. estimated measles antibody prevalence in children pre- (n = 1928, age 9 months to 5 years) and post-vaccination (n = 745, age 9 months to 19 years) campaign (17). Measles antibodies were tested in oral fluids (ORACOL device, MMD, Worcester, UK) using a commercial ELISA kit (Enzygnost rubeolla (measles) IgG, Dade Behring Diagnostics, Marburg, Germany) with a 91.7% sensitivity and 91.9% specificity. Not only did this work evaluate the effectiveness of the vaccine campaign, it also identified specific age groups of concern which may be more susceptible to continued measles transmission post-vaccination. Therefore, oral fluid surveys have the capacity to inform healthcare experts for continued improvement of vaccination strategy.

The convenience of using saliva in ELISA-based antibody tests have many potential advantages over using serum in a public healthcare setting. Most notably, saliva can theoretically be collected by the patients without the need of a phlebotomist. This would potentially lead to a decrease in demand for medical personnel and alleviate some of the strain put on the healthcare system. OraSure Technologies, Inc. has already manufactured an oral antibody collection device (OACD) that meets EUA requirements and can be self-administered under healthcare worker guidance, making it useful when available phlebotomists are limited (18). Furthermore, this advantage could reduce the risk of exposure to healthcare workers, which was a concern when collecting whole blood for testing from patients during the pandemic. OraSure Technologies has also developed their own, ELISA test for SARS-CoV-2 that pairs with their OACD and has been demonstrated to have 90.9% sensitivity and 100% specificity, showing that saliva can be as useful as serum in ELISA-based antibody tests.

As newer variants of SARS-CoV-2 are emerging and vaccines are being administered to the public, easily accessible antibody tests within communities would be a useful strategy for monitoring the protection against infection and severe disease. Communities that have a lower prevalence of antibodies against SARS-CoV-2 are at risk of an outbreak, especially as the more transmissible B.1.617.2 variant continues to spread among unvaccinated communities (19). Areas where access to healthcare is limited would benefit from a more convenient mail-in service for receiving and sending self-administered saliva collection devices that can readily be mailed back to the provider for analysis.

With continued efforts to roll out vaccines, tracking antibody levels in the population through saliva-based assays can be useful for identifying vulnerable populations. In a cross-sectional study conducted by Weill Cornell Medicine and Amsterdam Infection and Immunity Institute, 53 healthcare workers who received the Pfizer vaccine and 13 healthcare workers who received the Moderna vaccine were tested for IgG and IgA antibodies against the RBD and S protein antigens in paired serum and saliva samples. The participants were then tested for antibody production for both specimens at time points between 1 – 2 weeks after administration of the first and second dose. Antibodies against the SARS-CoV-2 RBD were detectable in all 66 saliva and serum samples. Likewise, the levels of IgG in both saliva and serum remained detectable the longest (20).

## Discussion 

Given the persistence of SARS-CoV-2 and the need to roll out a massive amount of vaccine doses, tracking immunity in the population will be challenging. Therefore, a reliable, convenient, and scalable antibody detection method is needed to track antibody prevalence in the population. To keep up with this demand, ELISA-based antibody detection methods offer a practical solution. Biotechnological innovations have allowed for the adaptation of reliable tests to more easily collected specimen types, further advancing the accessibility of ELISA-based antibody detection. Gold standard serum-based antibody detection is not as accessible nor scalable compared to saliva-based assays due to the invasiveness of the sample type and the requirement of trained professionals for sample collection. Furthermore, saliva specimens remain stable longer at ambient temperatures, allowing for more practical shipment of samples where needed.

Together with the ease of scaling and potential for use with saliva specimen types, ELISAs stand out as the optimal analytical tool for population serosurveillance, especially as vaccines are being distributed as the primary measure to stop the spread of SARS-CoV-2. These unique attributes provide a reliable and convenient way to track immunity worldwide.

## Author Contributions

JGC, ANA, MAM, RL and CB identified resources and drafted the review manuscript and designed and drafted the figures. MLB drafted, compiled, and edited the review manuscript and figures. SD oversaw the compilation and edited the manuscript. PC edited the manuscript. FT, VS and AI conceptualized the project, oversaw the review compilation and edited the manuscript. All authors contributed to the article and approved the submitted version.

## Conflict of Interest

All authors at the time of the research are, or were employed by Curative, a COVID-19 diagnostic company. FT and VS have partial ownership of Curative.

## Publisher’s Note

All claims expressed in this article are solely those of the authors and do not necessarily represent those of their affiliated organizations, or those of the publisher, the editors and the reviewers. Any product that may be evaluated in this article, or claim that may be made by its manufacturer, is not guaranteed or endorsed by the publisher.

## Supplementary Material

Supplementary Figure 1 Schematic of an indirect ELISA. **(A)** An antigen, like the spike protein from the SARS-CoV-2 Virus, is coated at the bottom of a polystyrene well in a microtiter plate. **(B)** A sample (serum in this example) is added to the wells that are coated with the antigen and incubated. Any antibodies against that antigen that the patient’s immune system has produced will bind. **(C)** Once the patient’s antibodies have bound to the antigen, the wells are rinsed to reduce nonspecific binding. The secondary antibodies are then added and incubated. The secondary antibodies are linked to an enzyme and bind to the patient’s antibodies. **(D)** The wells are rinsed again, and a substrate is added. **(E)** The enzyme that is linked to the secondary antibody acts as a catalyst and reacts with the substrate that causes the solution in the wells to change colors. A stop solution is added to stop the reaction and prevent further color change. A spectrophotometer is used to read the signal at a that is produced from the reaction at a set wavelength from the bottom of each well. The antibodies that the patient produced will cause more capturing of the secondary antibody and thus produce a greater reaction with the substrate and thus a more intense coloration of the wells.
