# Supplementary figures and images for: Saliva-Based ELISAs for Effective SARS-CoV-2 Antibody Monitoring in Vaccinated Individuals
Source: Front Immunol. 2021 Sep 3;12:701411. doi: 10.3389/fimmu.2021.701411 (PMC8446671; doi:10.3389/fimmu.2021.701411)

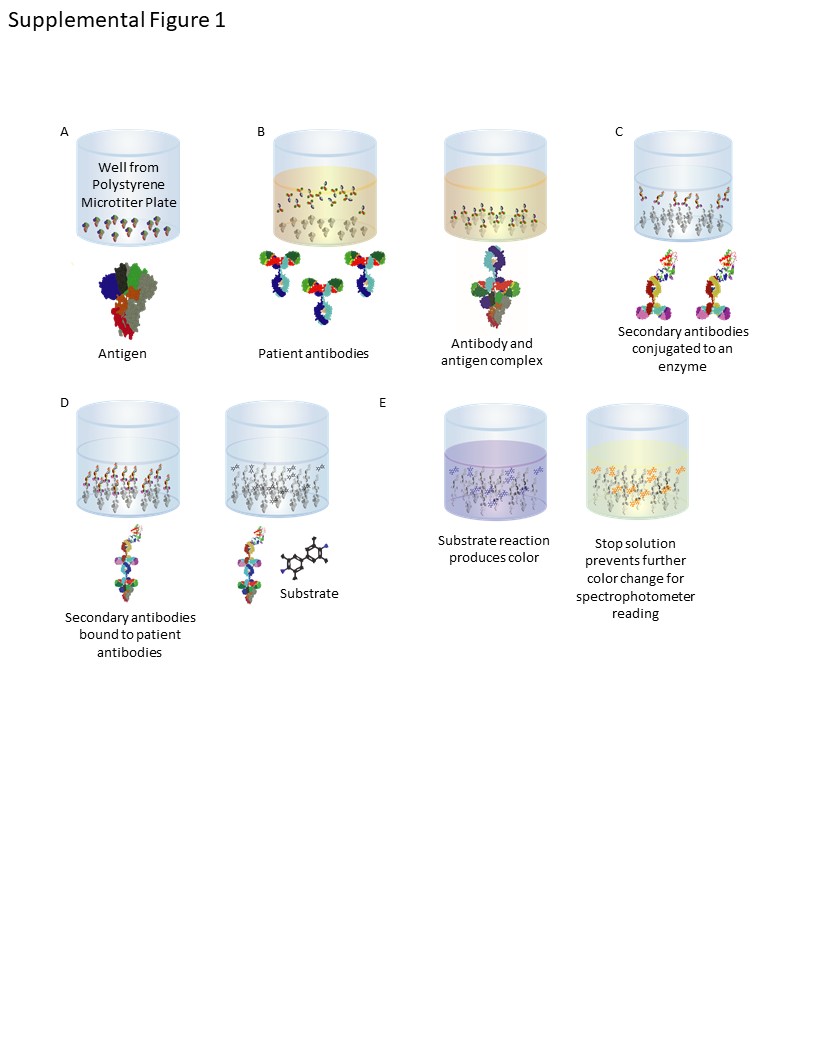

Supplement: Supplementary Figure 1 — Schematic of an indirect ELISA. (A) An antigen, like the spike protein from the SARS-CoV-2 Virus, is coated at the bottom of a polystyrene well in a microtiter plate. (B) A sample (serum in this example) is added to the wells that are coated with the antigen and incubated. Any antibodies against that antigen that the patient’s immune system has produced will bind. (C) Once the patient’s antibodies have bound to the antigen, the wells are rinsed to reduce nonspecific binding. The secondary antibodies are then added and incubated. The secondary antibodies are linked to an enzyme and bind to the patient’s antibodies. (D) The wells are rinsed again, and a substrate is added. (E) The enzyme that is linked to the secondary antibody acts as a catalyst and reacts with the substrate that causes the solution in the wells to change colors. A stop solution is added to stop the reaction and prevent further color change. A spectrophotometer is used to read the signal at a that is produced from the reaction at a set wavelength from the bottom of each well. The antibodies that the patient produced will cause more capturing of the secondary antibody and thus produce a greater reaction with the substrate and thus a more intense coloration of the wells. [file Image_1.jpg]
